# Validation of the Brazilian Version of the Modified Scale for Delineating Advanced Practice Nursing Roles

**DOI:** 10.1590/0034-7167-2023-0211

**Published:** 2024-07-19

**Authors:** Kamila Caroline Minosso, Mauricio Bedim dos Santos, Beatriz Rosana Gonçalves de Oliveira Toso

**Affiliations:** I Universidade Estadual do Oeste do Paraná. Cascavel, Paraná, Brazil; II Universidade Federal do Paraná. Toledo, Paraná, Brazil

**Keywords:** Nursing, Advanced Practice Nursing, Primary Health Care, Validation Studies, Public Health Nursing, Enfermería, Enfermería de Práctica Avanzada, Atención Primaria de Salud, Estudio de Validación, Enfermería en Salud Pública

## Abstract

**Objectives:**

to validate the Brazilian version of the Modified Scale for Delineating Advanced Practice Nursing Roles.

**Methods:**

this was a methodological study for the clinical validation of an instrument, conducted with 207 nurses working in primary care. Exploratory and confirmatory factor analysis, Cronbach’s alpha test, and z-test for proportion comparison were used.

**Results:**

the internal reliability of the scale was 0.944, with alpha greater than 0.80 in most domains, except for Education (0.786). In the exploratory factor analysis, considering the criterion of eigenvalue greater than one, eight factors were identified, explaining 79.38% of the variance. In the comparison of proportions, the adequate responses (≥ 2) in the domain of Comprehensive Direct Care, in both analyzed groups, were statistically equal. This domain had the highest score of adequate responses, followed by Education and Systems Support. Insufficient scoring was observed in the domains of Publication and Professional Leadership.

**Conclusions:**

the instrument demonstrated stability and reliability to be used in the evaluation of advanced nursing practice.

## RESUMEN

**Objetivos:**

validar la Escala Modificada de Delimitación de la Función del Enfermero de Prácticas Avanzadas, versión brasileña.

**Métodos:**

estudio metodológico de validación clínica de un instrumento, realizado con 207 enfermeros activos en la atención primaria. Se utilizaron análisis factorial exploratorio y confirmatorio, prueba alfa de Cronbach y prueba z para la comparación de proporciones.

**Resultados:**

la fiabilidad interna de la escala fue de 0,944, y el alfa fue mayor que 0,80 en la mayoría de los dominios, excepto en Educación (0,786). En el análisis factorial exploratorio, considerando el criterio de autovalor mayor que uno, se identificaron ocho factores, explicando el 79,38% de la varianza. En la comparación de proporciones, las respuestas suficientes (≥ 2) en el dominio de Cuidados Integrales Directos, en ambos grupos analizados, fueron estadísticamente iguales. Este dominio tuvo la mayor puntuación de respuestas suficientes, seguido por Educación y Apoyo de Sistemas. Se observó una puntuación insuficiente en los dominios de Publicación y Liderazgo Profesional.

**Conclusiones:**

el instrumento demostró estabilidad y fiabilidad para ser utilizado en la evaluación de la práctica avanzada de enfermería.

## INTRODUCTION

In the pursuit of adhering to strategies for implementing access to health care and resolving care issues, as recommended by the World Health Organization, the contribution and potential advancement of Advanced Practice Nursing (APN) in Primary Health Care (PHC) stands out. This approach yields positive results in health promotion, recovery, and disease prevention in less developed areas^(1)^, in addition to contributing to improved quality, efficiency, and sustainability^(2)^.

The role of nurses varies from country to country, exhibiting distinct competencies. Despite this, countries have adopted new strategies for training advanced practice nurses, from enhancing their competencies to solve increasingly complex health problems to defining medication treatments, backed by clinical protocols^(3)^.

The expanded professional practice differs from that of the nurse in PHC, due to the degree of autonomy in decision-making and in diagnosing and treating individual health problems^(4)^. Thus, these professionals have acquired increasing responsibilities in attending to users with chronic conditions, acting as vital allies in the evolution of treatment through regular exams, care provision, and continuous support^(5)^.

Nurses trained in advanced practice are associated with better patient survival rates^(2)^ and bring benefits to the countries that adopt them. Scientific evidence demonstrates their impact on services and health care costs, as these professionals deliver high-quality and safe services, aiding in the reduction of health care costs^(6)^.

Strategies for implementing APN in Latin America and the Caribbean, focusing on PHC, can be structured according to the specific characteristics of each country. Countries like Brazil and Chile have nursing master’s programs with great potential for the implementation of APN^(1)^. Although international experiences allow for the identification of different stages of APN development in various countries, in Brazil this function is still in its early stages, within the scope of discussions for its implementation in the country^(7)^.

Regulations and legislation in various countries in the Latin America region are not favorable to APN. However, Brazil has a high probability of establishing it, as it already has structured foundations focused on the autonomy and relevance of nursing in the health scenario, such as the Law on Professional Exercise and the National Primary Care Policy (PNAB)^(8-9)^.

Furthermore, it is observed that its implementation is identified as a human resources strategy in health to improve the recruitment and retention of nurses in their areas of practice and to provide opportunities for career progression, as well as the development of the profession^(10)^.

In light of the above, there is an increasing interest in supporting strategies for the effective implementation of APN. Thus, evaluating the practices developed by nurses can contribute to the growth and formalization of this new function in the national territory. This evaluation can be carried out using instruments already developed for this purpose, such as the Modified Scale for Delineating Advanced Practice Nursing Roles (EMDF/EPA), developed in English and widely used to assess APN competencies, already validated in Spanish and Portuguese^(11)^.

However, it is necessary to expand efforts to analyze, in the work process of professionals, practices that could be considered APN competencies, using international parameters produced to assess the function in countries where it occurs regularly.

## OBJECTIVES

To validate the Brazilian version of the Modified Scale for Delineating Advanced Practice Nursing Roles (EMDF/EPA).

## METHODS

### Ethical Aspects

The study was approved by the Research Ethics Committee. Informed consent was obtained from all individuals involved. The response to the form indicated consent to participate, as proceeding required agreement by marking the informed consent term. The study received approval from the CEP.

### Design and Location of the Study

This methodological study originated from the master’s thesis of the first author and utilized a quantitative approach for the clinical validation of the EMDF/EPA. This instrument is intended to measure and evaluate experiences in the specified context of use, after undergoing stages of analytical verification and validation^(12)^.

Online invitations to participate were sent to various municipalities across the 27 Brazilian states. A random selection criterion was applied for choosing the municipalities, which were classified as urban, adjacent urban, and adjacent rural according to the classification of the Brazilian Institute of Geography and Statistics (IBGE). This aimed to represent the heterogeneity of nursing practices in PHC. Contact with professionals was made through the municipal health departments of the selected municipalities and the Regional Nursing Councils (Corens) of all states via direct mail to increase the recruitment of professionals for the research.

The municipality of Florianópolis/Santa Catarina (SC) was adopted as a comparison model in the analysis relative to the other states. This choice was made because this municipality has a regulated Permanent Commission for the Systematization of Nursing Care, allowing the creation and implementation of nursing protocols^(13)^.

Care protocols are regulated according to Federal Law No. 7.498/1986, which addresses the regulation of nursing practice^(14)^, and Resolution No. 358/2009 of the Federal Nursing Council (COFEN)^(15)^. This provides nurses in PHC with greater autonomy in their clinical practice with legal support, including activities that can be considered as APN.

### Population and Inclusion/Exclusion Criteria

For the statistical analysis, the number of cases for the 41 items of the tested instrument was considered, following the 5:1 ratio, which allows for adequate analyses according to the literature^(16)^. For clinical validation, a minimum sample of 200 participants was suggested^(17)^, with the actual sample obtained being 207 nurses.

Inclusion criteria were: working in PHC; being a nurse; having Portuguese as the usual first language; and possessing computer skills, as the instrument was self-administered and virtual. Exclusion criteria included: nurse preceptors, consultants, and others without a formal employment relationship with the health service.

### Study Protocol and Data Collection Instrument

The EMDF/EPA is based on the Modified Advanced Practice Nursing Role Delineation Tool^(18)^, developed and validated in Australia, and subsequently transculturally validated in Spain^(19)^. This tool was designed to represent EPA activities across five practice domains, each encompassing competencies integral to them: Comprehensive Direct Care (14 competencies), Systems Support (9 competencies), Research, Education, and Publication, and Professional Leadership (6 competencies each).

Permission to use the tool was obtained from the original authors, through email correspondence with Anne M. Chang from the Queensland University of Technology in Australia.

The results from the previous stage of transcultural validation, regarding construct and content, were developed by the authors^(11)^, achieving a Cronbach’s alpha of 0.98, considered excellent, and an intraclass correlation of 0.61, regarded as substantial. This indicated that the tool is culturally adapted for identifying the competencies of PHC nurses in the development of APN.

The EMDF/EPA can be self-administered or used in an interview format. It consists of two sections: Section A, pertaining to sociodemographic data, and Section B, focusing on APN activities, which includes 41 items covering the main practice areas.

Participants are required to indicate the amount of time spent on each activity, marking the corresponding item on a Likert scale, represented qualitatively as “4-Much time,” “3-Quite a bit of time,” “2-Some time,” “1-Little time,” “0-No time,” respectively. Scores range from 0 to 164 points. The overall score, which is the average of all items, and the score for each dimension, which is the average of the respective items, are calculated.

The same metric used in the Spanish version of the scale^(20)^ was adopted in this study. Although the Portuguese version of the instrument differs from the Spanish version due to transcultural validation, the content of the items remained consistent. Therefore, the average score for the domains is calculated by adding all the activity scores for each domain and dividing by the number of activities. As such, the minimum average score for each domain indicating advanced practice is: 2.0 for Comprehensive Direct Care, Systems Support, and Education, and 1.7 for Research, Publication, and Professional Leadership.

For data collection, an online form was developed using Google Forms, including sociodemographic data and the scale. It was distributed via email and/or WhatsApp groups to PHC nurses, and completed by those interested in participating who met the inclusion criteria. Each item required a response, except for the comments section. Data collection occurred from February to September 2021.

### Analysis of Results and Statistics

Data were compiled in a Microsoft Excel^®^ spreadsheet, automatically generated by the platform. Absolute and relative frequency measures were utilized to characterize the sample and the responses to the 41 instrument items. Analyses were conducted using IBM Statistical Package for the Social Sciences (SPSS) software version 27, with a significance level set at 5% (α= 0.05). To assess the adequacy of clinical validation, both exploratory and confirmatory factor analyses were conducted.

The Kaiser criterion, where the eigenvalue is the sum of the squared factor loadings of the items, indicating the variance each item can be explained by the factor^(21)^, was used to determine if the extracted factors were correlated. Additionally, a Scree Plot graph was employed to evaluate the inflection point in the curve’s slope.

Following the factor extraction, an Oblimin oblique rotation with Kaiser normalization was performed to explore correlations between the factors and items, setting the cutoff for factor loading at 0.3^(22)^. In the confirmatory factor analysis, the model fit was evaluated using Structural Equation Models in R software. The models tested were based on the exploratory analysis and the model defined by the scale’s authors in the English version^(18)^.

The maximum likelihood estimator (lm) was used in the analyses, with fit indices categorized as absolute, parsimonious, and comparative^(23)^. The fit indices employed were: SRMR (Standardized Root Mean Square Residual), RMSEA (Root-Mean-Square Error of Approximation)^(24-25)^, and TLI (Tucker-Lewis Index)^(26)^.

The appropriateness of factor analysis was verified through the Kaiser-Meyer-Olkin (KMO) test and Bartlett’s test of sphericity. The closer the results are to 1, the more reliable they are considered, with the minimum acceptable value being 0.6^(27-28)^.

Statistical comparison tests were conducted with the states yielding the highest number of responses, maintaining proportionality, including: Santa Catarina (comparison group), Rio Grande do Sul (RS), Rio de Janeiro (RJ), and Paraná (PR). It is noteworthy that although sociodemographic data were compared with the scale responses, no statistically significant association was found, and thus they were not presented.

The z-test was utilized for proportion comparisons to assess “sufficient” responses in each construct dimension between two groups: nurses active in the municipality of Florianópolis/SC and nurses active in the aforementioned Brazilian states.

For scale reliability, the Cronbach’s alpha test was employed, classified as: greater than 0.80 - almost perfect, from 0.80 to 0.61 - substantial, from 0.60 to 0.41 - moderate, from 0.40 to 0.21 - fair, and below that, small^(29)^.

## RESULTS

Responses to the forms were received from 15 states, including Bahia (n=3), Ceará (n=3), Distrito Federal (n=2), Mato Grosso (n=1), Minas Gerais (n=1), Paraíba (n=10), Paraná (n=41), Pernambuco (n=12), Piauí (n=1), Rio de Janeiro (n=23), Rio Grande do Norte (n=2), Rio Grande do Sul (n=21), Rondônia (n=5), Santa Catarina (n=79), and São Paulo (n=3).

Regarding sociodemographic data, the predominant age group was between 30 and 39 years (Florianópolis: 53.16%; other states: 42.96%), and in terms of gender, there was a predominance of females (89.97% and 96.09%, respectively).

In terms of the current role, the most prevalent position was that of a clinical/assistential nurse (Florianópolis: 70.89%; other states: 40.63%). The majority of nurses did not have more than one employment contract (Florianópolis: 92.41%; other states: 72.66%). Regarding weekly workload, the range of 30 to 40 hours worked in PHC was prominent in both groups (Florianópolis: 45.45%; other states: 35.00%).

Analyzing professional experience, in Florianópolis, the most common tenure was 6 to 10 years (25.32%), followed by 16 to 20 years (20.25%). In other states, it was 11 to 15 years (21.09%), followed by less than one year (19.53%) and 1 to 5 years (19.53%). The duration of experience in the current position for both groups was 1 to 5 years (Florianópolis: 29.11%; other states: 34.38%), followed by 6 to 10 years (Florianópolis: 34.18%; other states: 21.09%). The primary workplace in both groups was Florianópolis (94.94%) and the other states (78.13%).

Regarding the education level of participants, the most cited highest level was specialization for both groups (Florianópolis: 39.24%; other states: 75.95%). Specialization in public/community health was most commonly mentioned, both in Florianópolis (45.83%) and in the other states (36.42%), although master’s degrees were also noted.

The internal reliability of the scale was 0.94. Across its five domains, the Cronbach’s alpha values were greater than 0.83, with 0.92 for the Comprehensive Direct Care domain, followed by 0.83 for Systems Support, 0.82 for Education, 0.86 for Research, and 0.91 for Publication and Professional Leadership.

Regarding the KMO test, the result was 0.90, indicating that the sample size was adequate. As for Bartlett’s test of sphericity, for factor analysis to be considered adequate, the test needs to indicate statistical significance, which was achieved in this study (χ^2^= 271.39; p-value <0.0001).

**Figure 1:**
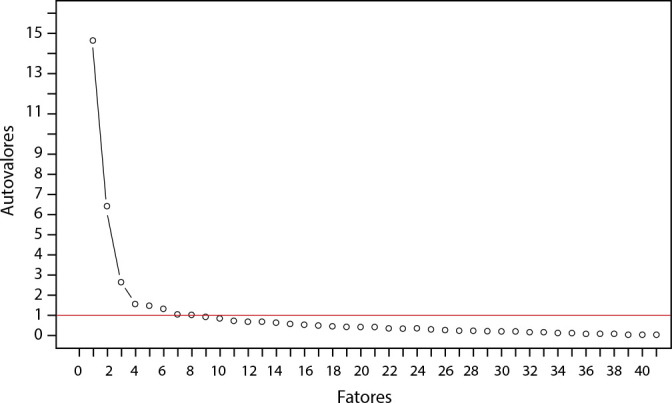
Scree Plot of the Eigenvalues Extracted by the Principal Component Analysis of the Modified Scale for Delineating Advanced Practice Nursing Roles

In the exploratory factor analysis, considering the criterion of an eigenvalue greater than one, eight factors were identified, explaining 79.38% of the variance, as presented in Table 1. This was corroborated by the Scree Plot, which showed a change in the slope’s inclination from the largest to the smallest eigenvalue (Figure 1).

**Table 1:** Eigenvalues, Explained Variance, and Cumulative Explained Variance of the First 15 Components Extracted by the Principal Components Analysis for the Modified Scale for Delineating Advanced Practice Nursing Roles

Component	Eigenvalue	Explained Variance	Cumulative Explained Variance
1	14.63	35.68%	35.68%
2	6.41	15.64%	51.32%
3	2.65	17.01%	68.33%
4	1.56	2.52%	70.85%
5	1.47	2.44%	73.29%
6	1.30	2.28%	75.57%
7	1.03	2.04%	77.61%
8	1.00	1.77%	79.38%

To confirm the hypothesis of eight dimensions of the scale and to seek the best fit, the results of the Principal Components Analysis were subjected to an Oblimin (OBLIMIN) rotation. The values of the factor loadings and shared variance (communalities) are presented in Table 2.

**Table 2:** Factor Loading Matrix of the Rotated Components of the Modified Scale for Delineating Advanced Practice Nursing Roles

Question (Dimension)	Components								
	1	2	3	4	5	6	7	8	h^2^*^^
4.3(4) Contributes to the identification of potential funding sources for the development and implementation of clinical projects/programs.	0.33								0.49
5.1(5) Disseminates nursing knowledge through presentations or publications at local, regional, national, and international levels.	0.47								0.59
5.2(5) Acts as a resource or member of committees in professional organizations.	0.70								0.66
5.3(5) Serves as a consultant for individuals and professional or lay groups and other hospitals/institutions.	0.52								0.53
5.4(5) Represents nursing in institutional or community forums focused on the educational needs of various population groups.	0.96								0.88
5.5(5) Represents the image of the nursing professional in institutional and/or community forums.	0.89								0.82
5.6(5) Collaborates with other healthcare professionals to provide leadership in formulating public health policies.	0.64								0.68
1.3(1) Identifies and requests diagnostic tests and procedures.		0.43							0.43
1.4(1) Gathers and interprets assessment data to formulate a care plan.		0.59							0.61
1.6(1) Assesses patient/family response to the proposed treatment and modifies the care plan based on the response.		0.96							0.88
1.7(1) Communicates the care plan and responses to the patient/family.		0.70							0.66
1.10(1) Acts as a consultant (expert guidance) in improving patient care and nursing practice based on area of expertise.		0.32							0.28
1.11(1) Facilitates the ethical decision-making process in patient care.		0.45							0.55
2.7(2) Acts as a mentor/tutor/preceptor/guide.			0.84						0.75
3.2(3) Serves as an educator and clinical preceptor for nursing and/or medical students, staff, and/or others.			0.95						0.92
2.2(2) Contributes to, consults on, or collaborates with other healthcare professionals in recruitment and retention activities (monitoring the professional’s clinical practice over a certain period for definitive hiring and/or approval in a public service probationary stage).				0.51					0.49
2.3(2) Participates in the strategic planning of the service, department, management, hospital directorate, or health system.				0.77					0.64
2.4(2) Directs and participates in quality improvement programs for the unit/service.				0.90					0.78
2.5(2) Actively participates in the development, implementation, and evaluation of quality improvement programs in collaboration with nursing leadership.				0.83					0.74
2.6(2) Demonstrates leadership in developing, implementing, and evaluating practice protocols, policies, and procedures.				0.64					0.58
4.1(4) Conducts clinical research.					0.76				0.61
4.2(4) Participates in research to monitor and improve the quality of care practices.					0.91				0.87
1.12(1) Coordinates interdisciplinary/interprofessional care plans for patients.		0.31				0.34			0.52
1.13(1) Collaborates with other services to optimize the patient’s health status.						0.49			0.58
1.14(1) Facilitates patient flow among various healthcare system services.		0.32				0.44			0.56
2.1^**^(2) Consults with other professionals regarding the conduct of projects and presentations.		0.20	0.23	0.24	0.21	0.26			0.46
3.1^**^(3) Evaluates ongoing education programs and recommends revisions when necessary.	0.20	0.20				0.25			0.53
3.3(3) Identifies learning needs of various population groups and contributes to the development of educational programs and resources.	0.32		0.30			0.44			0.69
3.5(3) Facilitates the professional development of the nursing team through continuing/permanent education.						0.37		0.35	0.68
3.6(3) Provides appropriate education to patients and families.						0.36			0.48
1.1(1) Performs and documents patient history and physical examination.							0.62		0.59
1.2(1) Assesses psychosocial, cultural, and religious factors that interfere with patient needs.		0.39					0.40		0.47
1.5(1) Performs specific care and procedures.							0.46		0.43
1.8(1) Promotes health education (counseling) to patients/families.							0.46		0.57
1.9(1) Adequately documents in the patient’s medical record.							0.73		0.66
2.8(2) Advocates for the role of nursing.								0.36	0.34
2.9(2) Acts as a spokesperson for nursing and the service when interacting with other professionals, patients, and the general public.								0.44	0.41
3.4(3) Serves as an educator for the team during direct care activities.								0.39	0.46
4.4(4) Uses research and integrates theory into practice, recommending changes in strategies/policies based on research.						0.31		0.43	0.58
4.5(4) Identifies clinical data that need to be collected and which are available in information systems/medical records for nursing and obstetrics research, as well as for quality assurance projects.								0.53	0.63
4.6(4) Collaborates with Information Specialists in designing information systems for research projects and quality assurance in nursing and obstetrics.								0.36	0.56

* h2 - Shared Variance;

** Factor Loading < 0.3.

In Table 2, factor loadings below 0.3 were omitted to clearly indicate which items belong to each factor based on higher factor loadings. It is noteworthy that two questions with factor loadings below 0.3 (Q2.1 - D2 and Q3.1 - D3) were retained, and their factor loadings were divided among several components.

In the confirmatory analysis stage, two models were tested: the first model considering the five dimensions proposed by the authors of the scale in the English version^(18)^, referred to as Model 1, and the second model with the eight dimensions obtained in the exploratory analysis, called Model 2. The quality indices of the models’ fit to the data of the Brazilian sample were as follows: Model 1: RMSEA-0.09; SRMR-0.11; TLI-0.74, while for Model 2, they were: RMSEA-0.05; SRMR-0.03; TLI-0.90. Model 2 achieved a better fit compared to Model 1 (RMSEA<0.06).

For the TLI index, according to the literature, values between 0.90 and 0.95 indicate a good model fit, and values less than 0.9 suggest considerations for model rejection^(30-31)^. The value obtained for Model 2 was 0.9, showing adequacy. Table 3 presents the comparison of proportions between the domains, comparing Florianópolis to the other states.

**Table 3:** Z-test for Differences in Proportions

Dimension	Florianopolis (Santa Catarina) (n= 79)			Other States (n= 128)			*p* value
	Suficiente	Total	Proporção	Suficiente	Total	Proporção	
Comprehensive Direct Care	659	1106	0.596	1038	1792	0.579	0.377
Systems Support	248	711	0.349	602	1152	0.523	<0.001
Education	175	474	0.369	347	768	0.452	0.004
Research	72	474	0.152	240	768	0.313	<0.001
Publication and Professional Leadership	45	474	0.095	176	768	0.229	<0.001
General	1199	3239	0.370	2403	5248	0.458	<0.000

The differences in the proportions of adequate responses between Florianópolis and the other states for the Comprehensive Direct Care domain were statistically equal (*p* value 0.377), with Florianópolis at 0.596 and the other states at 0.579. This same domain stands out in both groups for receiving more time dedication. For the domains of Systems Support, Education, Research, Publication, and Professional Leadership, statistically significant differences were found (*p* value <0.05), with the proportion of adequate responses from Florianópolis being lower when compared to those of the other states.

Table 4 describes the average scores obtained in each domain, representing the activities developed by the nurses.

**Table 4:** Evaluation of APN Performance by Nurses (N = 207)

Dimension	All States		Santa Catarina		Rio de Janeiro		Paraná		Rio Grande do Sul	
	Average	% ≥ a 2	Average	% ≥ a 2	Average	% ≥ a 2	Average	% ≥ a 2	Average	% ≥ a 2
General	2.1	60.4%	2.0	55.7%	2.4	78.3%	2.2	56.1%	2.2	57.1%
Comprehensive Direct Care	2.7	82.6%	2.7	87.3%	2.7	82.6%	2.6	80.5%	2.5	81.0%
Systems Support	2.2	59.4%	2.0	50.6%	2.4	69.6%	2.3	61.0%	2.3	66.7%
Education	2.2	60.9%	2.0	57.0%	2.6	87.0%	2.2	51.2%	2.2	61.9%
Research	1.7	39.1%	1.4	24.1%	2.3	78.3%	1.7	36.6%	1.8	42.9%
Publication and Professional Leadership	1.2	25.6%	0.9	16.5%	1.8	47.8%	1.3	29.3%	1.4	28.6%

An average score greater than 2.0 was observed in the domains of Comprehensive Direct Care, Systems Support, and Education. An average score of 1.7 occurred in the Research domain. The exception was the Publication and Professional Leadership domain (1.2), which did not achieve the minimum score. Rio de Janeiro (RJ) showed higher scores in all domains when compared to other states (SC, PR, and RS).

The domain with the highest score in the states was Comprehensive Direct Care (82.6%), followed by Education (60.9%) and Systems Support (59.4%). The Publication and Professional Leadership domain, which scored 1.2, cannot be considered a competency for APN as performed by the sample.

## DISCUSSION

The factor analysis of the original scale, which consists of five domains, was retained in this study, enabling the successful clinical validation of the Modified Scale for Delineating Advanced Practice Nursing Roles (EMDF/EPA). In examining APN activities within the Brazilian context, it was observed that nursing professionals engage in activities compatible with APN, as an average response (≥ 2) was recorded for most domains, with the exceptions being Research, Publication, and Professional Leadership. However, no participant fulfilled all the criteria to be considered an APN.

In characterizing the study’s participants, women, predominantly aged between 30 and 39 years, holding an employment contract, and having specialization as the highest level of education, were the majority. The sociodemographic data of this study align with those from the study conducted in Australia to validate the Modified Advanced Practice Role Delineation (APRD) Tool^(18)^. The only difference was in professional experience time: in Florianópolis, it ranged from 6 to 10 years, while in other states, it varied from 11 to 15 years. The duration of experience in the current role was 1 to 5 years for both groups analyzed, in contrast to the average experience time of 22.34 years and in the current role of 6.06 years, as noted in the aforementioned study.

Our study focused on nurses working in Primary Health Care (PHC), with the predominant roles being clinical/assistential nurse and unit coordinator/team supervisor. In other studies using the same scale, professionals from various health care settings participated^(18,32)^, indicating that the scale is applicable for evaluating advanced practice in any context, a hypothesis that still needs to be tested in Brazil.

According to the International Council of Nurses (ICN), one of the prerequisites for APN practice is a professional master’s degree with specialized training in the nurse’s field^(33)^. In our study, of the 207 responding nurses, 56 (27.05%) held various master’s degrees; however, there was no significant correlation between the highest level of education and the practice of APN.

In comparison to the validation conducted in Spain^(32)^, the exploratory factor analysis there yielded a matrix of six main factors, one more than the original scale, accounting for 63.72% of the total variance. This differs from our findings, where eight factors were identified. For the internal consistency analysis, reliability was assessed using the Cronbach’s alpha coefficient, with results for each of the six domains being: specialized care planning: 0.861, comprehensive care: 0.917, interprofessional collaboration: 0.838, training/education: 0.794, evidence-based practice and research: 0.899, and professional leadership: 0.888. The analysis indicated that domains 1 and 2 (specialized care planning and comprehensive care) scored the highest, while domains 5 and 6 (research and evidence-based practice and professional leadership) scored the lowest, paralleling our results.

The factor analysis of Chang et al.’s (2012)^(18)^ original scale revealed five factors, named according to the five domains of the original tool that inspired the instrument. Items 1-14, 27, and 29 were loaded onto Factor 2 (Comprehensive Direct Care); items 15-23 onto Factor 3 (Systems Support); items 25-28 onto Factor 4 (Education); items 30-35 onto Factor 5 (Research); and items 36-41 onto Factor 1 (Publication and Professional Leadership). The Cronbach’s alpha coefficient for the instrument was 0.94, with individual factors scoring as follows: Comprehensive Direct Care (α=0.95), Systems Support (α=0.93), Education (α=0.83), Research (α=0.90), and Publication and Professional Leadership (α=0.94). Our results were similar, confirming the scale’s validation.

The analysis using the EMDF/EPA tool indicates that the domain with the most “adequate” responses was Comprehensive Direct Care, while Publication and Professional Leadership were the least performed. This aligns with the literature^(1)^, which suggests that nurses invest less time in leadership actions and publications about their practice. In the referenced study, the practice-related domain showed that nurses possess decision-making skills and autonomy in medication prescribing and nursing diagnosis. However, the Education domain appeared weaker concerning recognition and accreditation of APN.

The findings demonstrated that nurses primarily engage in comprehensive direct patient care, with statistically significant results in the state of RJ (0.618) and Florianópolis (0.596). It’s noteworthy that in both contexts, nurses operate based on care protocols, supported by the Law of Professional Exercise and PNAB^(14,34)^. An established nursing protocol enables more decisive and autonomous professional actions^(35)^.

A large-scale study in the Western Pacific region on nurses’ responsibilities in advanced roles showed that they are not limited to clinical tasks in hospital settings but are also equipped for roles in PHC, education/teaching, professional leadership, quality management, and research^(36)^.

An integrative review described nurses as professionals with clinical competence for expanded nursing practice, possessing complex skills and functioning as clinicians, advisors, educators, protocol proposers, and researchers, with evidence-based practice^(37)^. In our results, nurses engaged in APN activities but did not dedicate sufficient time to activities in the domains of Research, Publication, and Professional Leadership.

Another study on advanced access in Basic Health Units (UBS) revealed that nurses were effective in 87.7% of consultations, exceeding the percentage suggested by the Ministry of Health for PHC service resolution^(38)^. However, it should be noted that clinical care alone does not define an APN; it is the combination of actions integrating the evaluated domains, with nurses making a difference in care by incorporating prevention and health promotion, comprehensive, and humanized care into their daily practice.

Therefore, there is a need to negotiate the expansion of practice and supportive legislation in the MS context, to ensure nurses can fully engage in care as per their professional preparation, contributing to the fulfillment of health policies^(1)^.

Furthermore, in the study conducted in Spain, 269 nurses met the criteria to be APN^(32)^. However, in our study, we found nurses performing some activities compatible with APN, but no nurse had adequate responses in all measured domains to be considered advanced practice. Thus, it’s necessary for nursing councils and health service administrations to encourage and support changes in legislation and education, through specific professional master’s programs for APN training, to implement the practice in the country.

### Study limitations

This study presents several limitations that should be considered. Firstly, although the research was extensively disseminated across all Brazilian states and achieved the necessary sample size, the sample might not fully represent the entire nursing population. Secondly, the study was based on self-reported responses, which could introduce a social desirability bias. Additionally, the absence of participants in certain specific domains of APN, such as Research and Publication, suggests there might be an incomplete understanding of the entire scope. This implies that nurses who could meet the criteria for defining APN may not have been included in the study.

### Contributions to the Field of Nursing

By investigating nursing practices across various Brazilian states, our study contributes a more comprehensive perspective on the competencies and activities performed by these professionals, particularly in the areas of Comprehensive Direct Care and Systems Support. It underscores the current practices and identifies gaps in APN in Brazil. This research provides impetus for the formal recognition and more thorough integration of these professionals at different levels of the healthcare system. The findings establish a solid base for future research, which could delve deeper into the less performed areas identified, and investigate the reasons behind the existing gaps in APN in Brazil.

## CONCLUSIONS

Based on our findings, we can affirm that the items evaluated are homogeneous and that the scale consistently measures its intended objectives, thereby being suitable for assessing the occurrence of APN in Brazil. Having a validated scale for this purpose is crucial for advancing the state of the art in Brazilian nursing. It offers scientific evidence for the analysis of professional practice, aimed at generating content on the topic of APN, which is still emerging in the country.

Consequently, further research is recommended to identify these professionals and to broaden the scope of research to practice settings beyond Primary Health Care (PHC). Additionally, it is essential to motivate legislative bodies and nursing human resource training institutions to address the challenges in implementing APN.
