# Supplementary material for: Validation of the Brazilian Version of the Modified Scale for Delineating Advanced Practice Nursing Roles
Source: Rev Bras Enferm. 2024 Jul 19;77(2):e20230211. doi: 10.1590/0034-7167-2023-0211 (PMC11259439; doi:10.1590/0034-7167-2023-0211)
Supplement: Translation

# Translation: Validação da Versão Brasileira da Escala Modificada de Delineamento da Função de Enfermeiro de Práticas Avançadas

**DOI:** 10.1590/0034-7167-2023-0211pt

**Authors:** Kamila Caroline Minosso, Mauricio Bedim dos Santos, Beatriz Rosana Gonçalves de Oliveira Toso

**Affiliations:** I Universidade Estadual do Oeste do Paraná. Cascavel, Paraná, Brasil; II Universidade Federal do Paraná. Toledo, Paraná, Brasil

**Keywords:** Enfermagem, Prática Avançada de Enfermagem, Atenção Primária à, Saúde, Estudo de Validação, Enfermagem em Saúde Pública

## Abstract

**Objetivos:**

validar a Escala Modificada de Delineamento da Função do Enfermeiro de Práticas Avançadas, versão brasileira.

**Métodos:**

estudo metodológico de validação clínica de instrumento, realizado com 207 enfermeiros atuantes na atenção primária. Utilizaram-se análise fatorial exploratória e confirmatória, teste alfa de Cronbach e teste z para comparação de proporções.

**Resultados:**

a confiabilidade interna da escala foi de 0,944, e o *alfa* maior que 0,80 na maioria dos domínios, exceto Educação (0,786). Na análise fatorial exploratória, considerando o critério do autovalor maior que um, identificaram-se oito fatores, explicando 79,38% da variância. Na comparação de proporções, as respostas suficientes (≥ 2) no domínio Cuidados Abrangentes Diretos, em ambos os grupos analisados, foram estatisticamente iguais. Esse domínio teve a maior pontuação de respostas suficientes, seguido por Educação e Suporte de Sistemas. Pontuação insuficiente foi observada nos domínios Publicação e Liderança Profissional.

**Conclusões:**

o instrumento demonstrou estabilidade e confiabilidade para ser utilizado na avaliação da prática avançada de enfermagem.

## INTRODUÇÃO

Com o intuito de aderir às estratégias para implementação do acesso à saúde e resolutividade do cuidado, recomendadas pela Organização Mundial de Saúde, destaca-se a contribuição e o potencial avanço da Prática Avançada de Enfermagem (PAE) na Atenção Primária à Saúde (APS). Esta abordagem propicia resultados positivos na promoção, recuperação da saúde e prevenção de doenças em locais menos desenvolvidos^(1)^, além de contribuir para uma melhor qualidade, eficiência e sustentabilidade^(2)^.

A atuação dos enfermeiros difere de um país para outro, apresentando competências distintas. Apesar disso, os países têm adotado novas estratégias para a capacitação do enfermeiro de prática avançada, desde a potencialização de suas competências para resolver problemas de saúde cada vez mais complexos, até a definição do tratamento medicamentoso, respaldados em protocolos clínicos^(3)^.

O exercício profissional ampliado se diferencia daquele que desempenha o enfermeiro na APS, em função do grau de autonomia na tomada de decisões e no diagnóstico e tratamento dos problemas de saúde dos indivíduos^(4)^. Deste modo, esses profissionais têm adquirido responsabilidades crescentes no atendimento aos usuários portadores de condições crônicas, atuando como importantes aliados na evolução do tratamento, por meio de exames regulares, prestação de cuidados e apoio contínuo^(5)^.

Enfermeiros com formação em prática avançada estão associados a uma melhor sobrevida do paciente^(2)^, além de trazer benefícios para os países que as adotam. Evidências científicas demonstram o seu impacto nos serviços e nos custos de saúde, uma vez que os profissionais entregam um serviço de alta qualidade e segurança, auxiliando na redução dos custos da assistência à saúde^(6)^.

Estratégias de implementação de PAE na América Latina e no Caribe, focadas na APS, podem ser estruturadas conforme as características específicas de cada país. Países como o Brasil e o Chile possuem programas de mestrado em enfermagem com grande potencial para implementação de PAE^(1)^. Embora experiências internacionais permitam identificar diferentes estágios de desenvolvimento da PAE em vários países, no Brasil essa função está em seus primórdios, ainda no escopo das discussões para sua implementação no país^(7)^.

As regulamentações e legislações de diversos países na região da América Latina não são favoráveis à PAE. Entretanto, o Brasil tem grande probabilidade de estabelecê-la, pois possui alicerces já estruturados com foco na autonomia e relevância da enfermagem no cenário da saúde, como por exemplo, a Lei do Exercício Profissional e a Portaria Nacional da Atenção Básica (PNAB)^(8-9)^.

Ademais, observa-se que a sua implantação é identificada como estratégia de recursos humanos na saúde para melhorar o recrutamento e a retenção de enfermeiros em suas áreas de atuação e para proporcionar oportunidades de progressão na carreira, assim como o desenvolvimento da profissão^(10)^.

Diante do exposto, há cada vez mais interesse em apoiar estratégias para a implementação efetiva da PAE. Assim, avaliar as práticas desenvolvidas pelos enfermeiros poderá contribuir para o crescimento e formalização da nova função em território nacional. Essa avaliação pode ser realizada por meio de instrumentos já desenvolvidos com essa finalidade, como a Escala Modificada de Delineamento de Função do Enfermeiro de Práticas Avançadas (EMDF/EPA), desenvolvida em língua inglesa e amplamente utilizada para avaliar competências de PAE, já validada para o espanhol e o português^(11)^.

Contudo, faz-se necessário ampliar os esforços para analisar, no processo de trabalho dos profissionais, práticas que poderiam ser consideradas competências de PAE, utilizando-se de parâmetros internacionais, produzidos para avaliar a função nos países em que ela ocorre regularmente.

## OBJETIVOS

Validar a Escala Modificada de Delineamento da Função do Enfermeiro de Práticas Avançadas (EMDF/EPA), versão brasileira.

## MÉTODOS

### Aspectos Éticos

O estudo foi aprovado pelo Comitê de Ética em Pesquisa. O Consentimento Livre e Esclarecido foi obtido de todos os indivíduos envolvidos no estudo. A resposta ao formulário indicou o consentimento para participar, pois, para prosseguir com o preenchimento, era necessário concordar, assinalando o termo de consentimento livre e esclarecido. O estudo recebeu aprovação do CEP.

### Desenho e Local do Estudo

Trata-se de um estudo metodológico, originário da dissertação de mestrado da primeira autora, com abordagem quantitativa, para a validação clínica da EMDF/EPA. Este instrumento destina-se a medir e avaliar a experiência no contexto de uso especificado, após passar por etapas de verificação e validação analítica^(12)^.

Foram enviados convites online para participação a diferentes municípios dos 27 estados brasileiros. O critério de seleção aleatória foi aplicado para a escolha dos municípios, nas classificações urbano, urbano adjacente e rural adjacente, conforme a classificação do Instituto Brasileiro de Geografia e Estatística (IBGE), visando representar a heterogeneidade das práticas profissionais dos enfermeiros na APS. O contato com os profissionais foi feito por meio das secretarias municipais de saúde dos municípios selecionados e dos Conselhos Regionais de Enfermagem (Corens) de todos os estados, via mala direta, para aumentar a captação de profissionais para a pesquisa.

O município de Florianópolis/Santa Catarina (SC) foi adotado como modelo de comparação na análise em relação aos demais estados. Essa escolha deveu-se ao fato de que este município possui uma Comissão Permanente de Sistematização da Assistência de Enfermagem regulamentada, permitindo a criação e implementação de protocolos de enfermagem^(13)^.

Os protocolos de cuidados são regulamentados de acordo com a Lei Federal nº 7.498/1986, que trata da regulamentação do exercício da Enfermagem^(14)^, e com a Resolução do Conselho Federal de Enfermagem (COFEN) nº 358/2009^(15)^, proporcionando aos enfermeiros da APS maior autonomia em sua atuação clínica com respaldo legal, inclusive em atividades que podem ser consideradas de PAE.

### População e Critérios de Inclusão/Exclusão

Para a análise estatística, considerou-se o número de casos para os 41 itens do instrumento testado, seguindo a proporção de 5:1, que permite análises adequadas conforme a literatura^(16)^. Para validação clínica, sugere-se uma amostra mínima de 200 participantes^(17)^, sendo a amostra mínima de 205 e a obtida de 207 enfermeiros.

Os critérios de inclusão foram: trabalhar na APS; ser enfermeiro(a); ter o português como primeira língua usual; e possuir habilidades de informática, uma vez que o instrumento era auto preenchido e virtual. Os critérios de exclusão incluíram: enfermeiros(as) preceptores(as), consultores(as) e outros(as) sem vínculo de trabalho formal com o serviço de saúde.

### Protocolo do estudo e instrumento de coleta

A EMDF/EPA é baseada no instrumento *Modified Advanced Practice Nursing Role Delineation Tool* ^(18)^, desenvolvido e validado na Austrália e validado transculturalmente na Espanha^(19)^. A ferramenta foi construída representando as atividades da EPA em cinco domínios de prática, cada um contendo as competências que os integram: cuidado abrangente direto (14 competências), suporte de sistemas (9 competências), pesquisa, educação e publicação, e liderança profissional (6 competências cada um).

A permissão para utilizar a ferramenta foi obtida dos autores originais, através de contato via e-mail com a autora Anne M. Chang, da *Queensland University of Technology*/Austrália.

O resultado da etapa anterior de validação transcultural, quanto ao constructo e conteúdo, foi desenvolvido pelas autoras^(11)^, obtendo-se um *alfa* de Cronbach de 0,98, considerado excelente, e uma correlação intraclasse de 0,61, considerada substantiva. Isso mostrou que a ferramenta está adaptada culturalmente para identificar as competências dos enfermeiros da APS no desenvolvimento de PAE.

A EMDF/EPA pode ser autoaplicável ou utilizada na forma de entrevista. É composta por duas sessões: a Sessão A, relativa aos dados sociodemográficos; e a Sessão B, às atividades de PAE, na qual encontram-se 41 itens para abordar as principais áreas de prática.

Os participantes devem indicar quanto tempo gastam em cada atividade, marcando o item correspondente em uma escala Likert, representada qualitativamente por “4-Muito tempo”, “3-Bastante tempo”, “2-Algum tempo”, “1-Pouco tempo”, “0-Nenhum tempo”, respectivamente. A pontuação varia de 0 a 164 pontos. Obtém-se a pontuação global, que é a média de todos os itens, e a pontuação para cada dimensão, que é a média dos itens correspondentes.

A mesma métrica utilizada na versão espanhola da escala^(20)^ foi adotada neste estudo, pois, embora o instrumento em língua portuguesa difira da versão espanhola devido à validação transcultural, a permanência dos itens se manteve. Assim, a pontuação média para os domínios é calculada adicionando todas as pontuações das atividades de cada domínio e dividindo pelo número de atividades. Deste modo, a pontuação mínima média para cada um dos domínios que indicam prática avançada é: Cuidados abrangentes diretos, Suporte de sistema e Educação - 2,0 para cada domínio, respectivamente; Pesquisa, Publicação e Liderança profissional - 1,7 para cada domínio.

Para a coleta de dados, foi elaborado um formulário online na plataforma *Google Forms*, contendo os dados sociodemográficos e a escala. Ele foi enviado por e-mail e/ou grupos de WhatsApp aos enfermeiros da APS, sendo respondido pelos interessados em participar da pesquisa e que atendiam aos critérios de inclusão. Cada item era de resposta obrigatória, com exceção do espaço para comentários. A coleta de dados ocorreu no período de fevereiro a setembro de 2021.

### Análise dos resultados e estatística

Os dados foram agrupados em planilha eletrônica do Microsoft Excel^®^, gerados automaticamente pela plataforma. Utilizaram-se medidas de frequência absoluta e relativa na caracterização da amostra e das respostas dos 41 itens do instrumento. As análises foram realizadas usando o software IBM *Statistical Package for the Social Sciences* (SPSS) versão 27, e o nível de significância adotado foi de 5% (α= 0,05). Para verificar a adequação da validação clínica, empregou-se a análise fatorial exploratória e confirmatória.

Para verificar se os fatores extraídos estavam correlacionados entre si, adotou-se o critério de Kaiser, no qual o autovalor representa a soma das cargas fatoriais ao quadrado dos itens, indicando a variância que cada item pode ser explicado pelo fator^(21)^. Adicionalmente, utilizou-se o gráfico de *Scree Plot* para avaliar o ponto de inflexão da inclinação da curva.

Após a extração dos fatores, realizou-se a rotação oblíqua Oblimin com Normalização de Kaiser para explorar a correlação entre os fatores e os itens, sendo o ponto de corte para carga fatorial adotado de 0,3^(22)^. Na análise fatorial confirmatória, avaliou-se o ajuste do modelo utilizando o *Structural Equation Models* do *software* R. Os modelos testados foram definidos considerando a análise exploratória realizada e o modelo definido pelos autores da escala na versão em inglês^(18)^.

Nas análises, utilizou-se o estimador de máxima verossimilhança (lm), cujos índices de ajuste podem ser classificados em três categorias: ajuste absoluto, parcimonioso e comparativo^(23)^. Os índices de ajuste utilizados foram: SRMR (*Standardized Root Mean Square Residual*), RMSEA (*Root-Mean-Square Error of Approximation*)^(24-25)^ e TLI (*Tucker-Lewis Index*)^(26)^.

A adequabilidade da utilização da análise fatorial foi verificada por meio do teste de *Kaiser-Meyer-Olkin* (KMO) e do teste de esfericidade de Bartlett. Quanto mais próximo de 1, mais confiáveis tendem a ser os resultados, sendo o mínimo tolerável de 0,6^(27-28)^.

Os testes estatísticos de comparação foram realizados com os estados que obtiveram maior número de respostas, mantendo a proporcionalidade, dentre os quais: Santa Catarina (grupo de comparação), Rio Grande do Sul (RS), Rio de Janeiro (RJ) e Paraná (PR). Cabe ressaltar que foram comparados os dados sociodemográficos com as respostas da escala, embora sem associação estatisticamente significativa; assim, não foram apresentados.

Utilizou-se o teste z para comparação de proporções, a fim de avaliar as respostas classificadas como “suficientes” em cada dimensão do constructo entre os dois grupos: enfermeiros(as) atuantes no município de Florianópolis/SC e enfermeiros(as) atuantes nos estados brasileiros mencionados acima.

Para a confiabilidade da escala, empregou-se o teste *alfa* de Cronbach, classificando-se em: maior que 0,80 - quase perfeita, de 0,80 a 0,61 - substancial, de 0,60 a 0,41 - moderada, de 0,40 a 0,21 - razoável e abaixo disso, pequena^(29)^.

## RESULTADOS

Houve retorno dos formulários preenchidos por 15 estados, sendo eles: Bahia (n=3), Ceará (n=3), Distrito Federal (n=2), Mato Grosso (n=1), Minas Gerais (n=1), Paraíba (n=10), Paraná (n=41), Pernambuco (n=12), Piauí (n=1), Rio de Janeiro (n=23), Rio Grande do Norte (n=2), Rio Grande do Sul (n=21), Rondônia (n=5), Santa Catarina (n=79) e São Paulo (n=3).

Quanto aos dados sociodemográficos, a faixa etária predominante foi entre 30 e 39 anos (Florianópolis: 53,16%; demais estados: 42,96%), e quanto ao sexo, houve uma proeminência do feminino (89,97% e 96,09%, respectivamente).

Em relação à função atual, observou-se como predominante o(a) enfermeiro(a) clínico/assistencial (Florianópolis: 70,89%; demais estados: 40,63%). A maioria dos enfermeiros não possui mais de um vínculo empregatício (Florianópolis: 92,41%; demais estados: 72,66%). Quanto à carga horária semanal, destacam-se de 30 a 40 horas trabalhadas na APS em ambos os grupos (Florianópolis: 45,45%; demais estados: 35,00%).

Ao analisar a experiência profissional, em Florianópolis, o tempo mais comum foi de 6 a 10 anos (25,32%), seguido de 16 a 20 anos (20,25%). Nos demais estados, foi de 11 a 15 anos (21,09%), seguido de menos de um ano (19,53%) e de 1 a 5 anos (19,53%). O tempo de experiência no cargo atual para ambos os grupos foi de 1 a 5 anos (Florianópolis: 29,11%; demais estados: 34,38%), seguido de 6 a 10 anos (Florianópolis: 34,18%; demais estados: 21,09%). O local de trabalho predominante foi em ambos os grupos, Florianópolis (94,94%) e nos demais estados (78,13%).

Quanto à escolarização dos participantes, observou-se que o nível mais alto citado foi especialização, para ambos os grupos (Florianópolis: 39,24%; demais estados: 75,95%). A especialização em saúde pública/coletiva foi comumente citada pela maioria, tanto em Florianópolis (45,83%) quanto nos demais estados (36,42%), embora o mestrado também tenha sido mencionado.

A confiabilidade interna da escala foi de 0,94. Nos seus cinco domínios, os valores de *alfa* de Cronbach foram maiores que 0,83, sendo 0,92 para o domínio Cuidados Abrangentes Diretos, seguido de 0,83 para Suporte de Sistemas, 0,82 para Educação, 0,86 para Pesquisa e 0,91 para Publicação e Liderança Profissional.

Quanto ao teste KMO, o resultado foi de 0,90, indicando que o tamanho da amostra foi adequado. Em relação ao teste de esfericidade de Bartlett, para a análise fatorial ser considerada adequada, o teste precisa indicar significância estatística, o que foi alcançado neste estudo (χ^2^= 271,39; valor de p<0,0001).

Na análise fatorial exploratória, considerando o critério do autovalor maior que um, foram obtidos oito fatores com 79,38% da explicação da variância, conforme apresentado na Tabela 1. Isso foi corroborado pelo Scree Plot, que mostrou mudança na inclinação do maior para o menor autovalor (Figura 1).

**Tabela 1:** Autovalores, variância explicada e variância explicada acumulada dos 15 primeiros componentes extraídos pela análise de componentes principais para a Escala Modificada de Delineamento de Função do Enfermeiro de Práticas Avançadas

Componente	Autovalor	Variância Explicada	Variância Explicada Acumulada
1	14,63	35,68%	35,68%
2	6,41	15,64%	51,32%
3	2,65	17,01%	68,33%
4	1,56	2,52%	70,85%
5	1,47	2,44%	73,29%
6	1,30	2,28%	75,57%
7	1,03	2,04%	77,61%
8	1,00	1,77%	79,38%

**Figura 1:** *Scree Plot* dos autovalores extraídos pela análise de componentes principais da Escala Modificada de Delineamento de Função do Enfermeiro de Práticas Avançadas

Para confirmar a hipótese de oito dimensões da escala e, para buscar o melhor ajuste, os resultados da Análise dos Componentes Principais foram submetidos a rotação Oblíqua (OBLIMIN), cujos valores das cargas fatoriais e da variância compartilhada (comunalidades) estão apresentados da Tabela 2.

**Tabela 2:** Matriz de carga fatorial dos componentes rotacionados da Escala Modificada de Delineamento de Função do Enfermeiro de Práticas Avançadas

Questão (Domínio)	Componentes								
	1	2	3	4	5	6	7	8	h^2^*^^
4.3(4) Contribui para a identificação de potenciais fontes de financiamento para o desenvolvimento e implementação de projetos/programas clínicos.	0,33								0,49
5.1(5) Dissemina o conhecimento de enfermagem por meio de apresentação ou publicação em níveis local, regional, nacional e internacional.	0,47								0,59
5.2(5) Atua como recurso ou membro de comitês em organizações profissionais.	0,70								0,66
5.3(5) Atua como consultor para indivíduos e grupos profissionais ou leigos e outros hospitais/instituições.	0,52								0,53
5.4(5) Representa a enfermagem em fóruns institucionais ou da comunidade focados nas necessidades educacionais de vários grupos populacionais.	0,96								0,88
5.5(5) Representa a imagem do profissional de enfermagem em fóruns institucionais e/ou da comunidade.	0,89								0,82
5.6(5) Colabora com outros profissionais de saúde para prover liderança na formulação de políticas públicas de saúde.	0,64								0,68
1.3(1) Identifica e solicita exames diagnósticos e procedimentos.		0,43							0,43
1.4(1) Reúne e interpreta dados da avaliação para formular plano de cuidados.		0,59							0,61
1.6(1) Avalia resposta do paciente/família ao tratamento proposto e modifica o plano de cuidados de acordo com a resposta.		0,96							0,88
1.7(1) Comunica o plano de cuidados e as respostas ao paciente/família.		0,70							0,66
1.10(1) Atua como consultor (orientação especializada) na melhoria da assistência ao paciente e na prática de enfermagem com base na expertise da área de especialização.		0,32							0,28
1.11(1) Facilita o processo de tomada de decisão ética no atendimento ao paciente.		0,45							0,55
2.7(2) Atua como mentor/tutor/preceptor/orientador.			0,84						0,75
3.2(3) Atua como educador e preceptor clínico para estudantes de enfermagem e/ou medicina, equipe e/ou outros.			0,95						0,92
2.2(2) Contribui, consulta ou colabora com outros profissionais de saúde em atividades de recrutamento e retenção (acompanhamento da prática assistencial do profissional durante determinado período para contratação definitiva e/ou aprovação em estágio probatório de serviço público).				0,51					0,49
2.3(2) Participa no planejamento estratégico do serviço, departamento, gerência, diretoria hospital ou sistema de saúde.				0,77					0,64
2.4(2) Direciona e participa dos programas de melhoria de qualidade da unidade/serviço.				0,90					0,78
2.5(2) Participa ativamente do desenvolvimento, implementação e avaliação de programas de melhoria de qualidade em colaboração com a liderança de enfermagem.				0,83					0,74
2.6(2) Apresenta liderança no desenvolvimento, implementação e avaliação nos protocolos de práticas, políticas e procedimentos.				0,64					0,58
4.1(4) Conduz investigação clínica.					0,76				0,61
4.2(4) Participa da investigação para monitorar e melhorar a qualidade de práticas de cuidado.					0,91				0,87
1.12(1) Coordena plano interdisciplinar/interprofissional para o cuidado aos pacientes.		0,31				0,34			0,52
1.13(1) Colabora com outros serviços para otimizar o estado de saúde do paciente.						0,49			0,58
1.14(1) Facilita o fluxo do paciente entre diversos serviços do sistema de saúde.		0,32				0,44			0,56
2.1^**^(2) Consulta outros profissionais a respeito de condução de projetos e apresentações.		0,20	0,23	0,24	0,21	0,26			0,46
3.1^**^(3) Avalia programas de educação permanente e recomenda revisão quando necessário.	0,20	0,20				0,25			0,53
3.3(3) Identifica necessidade de aprendizagem de vários grupos populacionais e contribui para o desenvolvimento de programas e recursos educacionais.	0,32		0,30			0,44			0,69
3.5(3) Facilita o desenvolvimento profissional da equipe de enfermagem por meio da educação continuada/permanente.						0,37		0,35	0,68
3.6(3) Oferece educação adequada para pacientes e familiares.						0,36			0,48
1.1(1) Realiza e documenta o histórico e exame físico do paciente.							0,62		0,59
1.2(1) Avalia fatores psicossociais, culturais e religiosos que interferem nas necessidades dos pacientes.		0,39					0,40		0,47
1.5(1) Executa cuidados específicos e procedimentos.							0,46		0,43
1.8(1) Promove ações de educação em saúde (aconselhamento) ao paciente/família.							0,46		0,57
1.9(1) Documenta adequadamente no prontuário do paciente.							0,73		0,66
2.8(2) Defende o papel da enfermagem.								0,36	0,34
2.9(2) Atua como porta voz para a enfermagem e o serviço ao interagir com outros profissionais, pacientes e público geral.								0,44	0,41
3.4(3) Atua como educador para a equipe durante a realização das atividades de cuidado direto.								0,39	0,46
4.4(4) Usa a pesquisa e integra teoria à prática e recomenda mudanças nas estratégias/políticas baseada em pesquisa.						0,31		0,43	0,58
4.5(4) Identifica os dados clínicos que precisam ser coletados e quais estão disponíveis em sistemas de informação/prontuário para pesquisas de enfermagem e obstetrícia, assim como para projetos de garantia da qualidade.								0,53	0,63
4.6(4) Colabora com Especialistas em Informação na concepção de sistemas de informação para projetos de pesquisa e garantia de qualidade em enfermagem e obstetrícia.								0,36	0,56

* h^2^ - variância compartilhada;

** carga fatorial < 0,3.

Na Tabela 2 foram omitidos os valores de carga fatorial menores que 0,3, assim é possível verificar claramente quais itens pertencem a cada um dos fatores por meio das cargas fatoriais mais altas. Ressalta-se que foram mantidas duas questões que obtiveram cargas fatoriais menores que 0,3 (Q2.1 - D2 e Q3.1 - D3), e suas cargas fatoriais foram divididas entre vários componentes.

Na etapa da análise confirmatória foram verificados dois modelos, o primeiro modelo considerando as cinco dimensões propostas pelos autores da escala na versão em inglês^(18)^, que será chamado de Modelo 1 e o segundo modelo com as oito dimensões obtidas na análise exploratória, denominado Modelo 2. Os índices de qualidade do ajuste dos modelos aos dados da amostra brasileira foram os seguintes: Modelo 1: RMSEA-0,09; SRMR-0,11; TLI-0,74, enquanto no Modelo 2, foi de: RMSEA-0,05; SRMR-0,03; TLI-0,90. O modelo 2 obteve um melhor ajuste quando comparado ao modelo 1 (RMSEA<0,06).

Para o índice TLI, conforme a literatura, valores entre 0,90 e 0,95 são indicativos de um bom ajuste do modelo e valores menores que 0,9 demonstram suspeitas para rejeição do modelo^(30-31)^. O valor obtido para o modelo 2 foi de 0,9, mostrando-se adequado. Na Tabela 3, apresenta-se a comparação de proporções entre os domínios, comparando-se Florianópolis aos demais estados.

**Tabela 3:** Teste z para diferenças de proporções

Domínios	Florianópolis (Santa Catarina) (n= 79)			Demais Estados (n= 128)			Valor de *p*
	Suficiente	Total	Proporção	Suficiente	Total	Proporção	
Cuidados Abrangentes Diretos	659	1106	0,596	1038	1792	0,579	0,377
Suporte de Sistemas	248	711	0,349	602	1152	0,523	<0,001
Educação	175	474	0,369	347	768	0,452	0,004
Pesquisa	72	474	0,152	240	768	0,313	<0,001
Publicação e Liderança Profissional	45	474	0,095	176	768	0,229	<0,001
Geral	1199	3239	0,370	2403	5248	0,458	<0,000

As diferenças de proporções de respostas suficientes entre Florianópolis e os demais estados, para o domínio Cuidados Abrangentes Diretos, foram estatisticamente iguais (*p* 0,377), sendo Florianópolis (0,596) e demais estados (0,579). Este mesmo domínio sobressai em relação aos demais, em ambos os grupos, com maior dedicação de tempo. Para os domínios Suporte de Sistemas, Educação, Pesquisa, Publicação e Liderança Profissional, verificou-se diferenças estatisticamente significativas (valor de *p*<0,05), sendo a proporção de respostas suficientes de Florianópolis menor quando comparada as proporções dos demais estados.

Na Tabela 4, estão descritas as médias obtidas em cada domínio, representando as atividades desenvolvidas pelos enfermeiros.

**Tabela 4:** Avaliação da realização de Prática Avançada de Enfermagem pelos enfermeiros (N = 207)

Dimensão	Todos os Estados		Santa Catarina		Rio de Janeiro		Paraná		Rio Grande do Sul	
	Média	% ≥ a 2	Média	% ≥ a 2	Média	% ≥ a 2	Média	% ≥ a 2	Média	% ≥ a 2
Geral	2,1	60,4%	2,0	55,7%	2,4	78,3%	2,2	56,1%	2,2	57,1%
Cuidados Abrangentes Diretos	2,7	82,6%	2,7	87,3%	2,7	82,6%	2,6	80,5%	2,5	81,0%
Suporte de Sistemas	2,2	59,4%	2,0	50,6%	2,4	69,6%	2,3	61,0%	2,3	66,7%
Educação	2,2	60,9%	2,0	57,0%	2,6	87,0%	2,2	51,2%	2,2	61,9%
Pesquisa	1,7	39,1%	1,4	24,1%	2,3	78,3%	1,7	36,6%	1,8	42,9%
Publicação e Liderança Profissional	1,2	25,6%	0,9	16,5%	1,8	47,8%	1,3	29,3%	1,4	28,6%

Média maior que 2,0 ocorreu nos domínios Cuidados Abrangentes Diretos, Suporte de Sistema e Educação. Média igual a 1,7 ocorreu no domínio Pesquisa. A exceção foi do domínio Publicação e Liderança Profissional (1,2) que não obteve a pontuação mínima. O RJ demonstra pontuações elevadas em todos os domínios quando comparado com os estados (SC, PR e RS).

O domínio com maior pontuação nos estados foi Cuidados Abrangentes Diretos (82,6%), seguido de Educação (60,9%) e Suporte de Sistemas (59,4%). O domínio Publicação e Liderança Profissional que obteve a pontuação de 1,2, não pode ser considerado competência para PAE desempenhado pela amostra.

## DISCUSSÃO

A análise fatorial da escala original, composta por cinco domínios, foi mantida neste estudo, o que nos permitiu realizar com sucesso a validação clínica da Escala Modificada de Delineamento de Função do Enfermeiro de Práticas Avançadas (EMDF/EPA). Na investigação das ações de PAE no contexto brasileiro, observou-se que os profissionais de enfermagem realizam atividades compatíveis com a EPA, visto que se obteve uma média de respostas (≥ 2) para a maioria dos domínios, exceto em Pesquisa e Publicação e Liderança Profissional. Contudo, nenhum participante preencheu todos os requisitos para ser considerado um EPA.

Na caracterização dos participantes do estudo, predominaram as mulheres, com idade entre 30 e 39 anos, possuindo um vínculo empregatício e tendo a especialização como nível mais alto de escolaridade. Os dados sociodemográficos deste estudo assemelham-se aos do estudo realizado na Austrália para validar a *Modified Advanced Practice Role Delineation* (APRD) Tool^(18)^. Apenas o tempo de experiência profissional diferiu: em Florianópolis foi de seis a 10 anos, enquanto nos demais estados variou de 11 a 15 anos. O tempo de experiência no cargo atual foi de um a cinco anos para ambos os grupos analisados, em contraste com o tempo médio de experiência de 22,34 anos e no cargo atual de 6,06 anos, conforme o estudo citado.

Nosso estudo focou em enfermeiros atuantes na APS, predominando a função de enfermeiro clínico/assistencial, seguida de enfermeiro coordenador de unidade/supervisor de equipe. Em outros estudos utilizando a mesma escala, participaram também profissionais que atuam em diferentes âmbitos de atenção à saúde^(18,32)^, demonstrando que a escala se aplica para avaliar a prática avançada em qualquer cenário de atuação, o que ainda precisa ser testado no Brasil.

De acordo com o *International Council of Nurses* (ICN), um dos requisitos para o exercício da EPA é o mestrado profissional com formação específica na área de atuação do enfermeiro^(33)^. No presente estudo, dos 207 enfermeiros respondentes, 56 (27,05%) possuíam mestrados diversos, porém, não houve associação significativa entre o mais alto nível de escolaridade e o exercício da EPA.

Em comparação à validação realizada na Espanha^(32)^, a análise fatorial exploratória naquele país obteve uma matriz de seis fatores principais, um a mais do que na escala original, explicando 63,72% da variância total, diferindo também de nossos resultados, onde foram encontrados oito fatores. Para a análise de consistência interna, a confiabilidade com o coeficiente *alfa* de Cronbach foi calculada, cujos resultados para cada um dos seis domínios foram: planejamento de cuidados especializados: 0,861, cuidado integral: 0,917, colaboração interprofissional: 0,838, treinamento/educação: 0,794, prática e pesquisa baseadas em evidências: 0,899 e liderança profissional: 0,888. A análise mostrou que os domínios 1 e 2 (planejamento de cuidados especializados e atenção integral) apresentaram as maiores médias, enquanto os domínios 5 e 6 (pesquisa e prática baseada em evidências e liderança profissional) apresentaram as menores, similar aos nossos resultados.

Os resultados da análise fatorial da escala original de Chang et al. (2012)^(18)^ mostraram que foram cinco os fatores, nomeados de acordo com os cinco domínios da ferramenta original que originou o instrumento. Os itens 1-14, 27 e 29 foram carregados no Fator 2 (atenção integral direta); itens 15-23 no Fator 3 (suporte de sistemas); itens 25-28 no Fator 4 (educação); itens 30-35 no Fator 5 (pesquisa) e os itens 36-41 no Fator 1 (publicação e liderança profissional). O coeficiente *alfa* de Cronbach para o instrumento foi de 0,94 e para cada um dos fatores: atenção integral direta (α=0,95), suporte dos sistemas (α=0,93), educação (α=0,83), pesquisa (α=0,90) e publicação e liderança profissional (α=0,94). Nossos resultados foram todos similares, confirmando a validação da escala.

A análise realizada com a ferramenta EMDF/EPA indica que o domínio com mais respostas “suficientes” foi Cuidados Abrangentes Diretos, enquanto o menos realizado foi Publicação e Liderança Profissional. Isso está em consonância com a literatura^(1)^, que aponta os enfermeiros investindo menos tempo em ações de liderança e publicações sobre sua prática. No estudo referido, o domínio relacionado à prática evidenciou que o enfermeiro possui habilidades para tomada de decisão e autonomia para prescrição de medicamentos e diagnóstico de enfermagem. Entretanto, o domínio educação mostrou-se mais frágil no que tange ao reconhecimento e credenciamento da EPA.

Os achados demonstraram que os enfermeiros atuam principalmente em cuidados abrangentes diretos ao paciente, sendo que no estado do RJ (0,618) e em Florianópolis (0,596) os resultados foram estatisticamente significativos. Vale ressaltar que, em ambas as realidades, os enfermeiros atuam com base em protocolos assistenciais de cuidado, respaldados na Lei do Exercício Profissional e PNAB^(14,34)^. Um protocolo de enfermagem instituído possibilita ações profissionais mais resolutivas e autônomas^(35)^.

Um estudo de grande porte desenvolvido na região do Pacífico Ocidental sobre as responsabilidades dos enfermeiros em funções avançadas mostrou que eles não estão limitados apenas às tarefas clínicas em ambientes hospitalares, mas também estão preparados para a atuação na APS, educação/ensino, liderança profissional, gestão da qualidade e pesquisa^(36)^.

Uma revisão integrativa descreveu os enfermeiros como profissionais com competência clínica para a prática expandida da enfermagem, possuindo habilidades complexas e atuando como clínicos, assessores, educadores, propositores de protocolos e pesquisadores, com a prática baseada em evidências^(37)^. Em nossos resultados, os enfermeiros desenvolveram atividades de PAE, mas não dedicaram tempo suficiente às atividades dos domínios Pesquisa, Publicação e Liderança Profissional.

Outro estudo sobre o acesso avançado na Unidade Básica de Saúde (UBS) evidenciou que o enfermeiro foi resolutivo em 87,7% das consultas, superando o percentual sugerido pelo Ministério da Saúde em relação à resolutividade do serviço da APS^(38)^. Entretanto, é preciso observar que somente o cuidado clínico não define um EPA, mas sim o conjunto de ações que integram os domínios avaliados, lembrando que o enfermeiro faz a diferença no cuidado justamente por incorporar em seu cotidiano ações de prevenção e promoção da saúde, cuidado integral e humanizado.

Portanto, vislumbra-se a necessidade de negociar a ampliação da prática e de legislações que respaldem esse profissional no contexto do MS, para assegurar aos enfermeiros uma atuação integral na assistência, conforme seu preparo profissional, contribuindo para o cumprimento das políticas de saúde^(1)^.

Ademais, no estudo realizado na Espanha, identificou-se que 269 enfermeiros preenchiam os requisitos para serem EPA^(32)^. Contudo, em nosso estudo, encontraram-se enfermeiros realizando algumas atividades compatíveis com aquelas desenvolvidas por EPA, porém nenhum enfermeiro teve suficiência de respostas em todos os domínios mensurados para ser considerado de prática avançada. Desse modo, é necessário que os conselhos de enfermagem e a administração dos serviços de saúde estimulem e apoiem mudanças na legislação e na educação, por meio de mestrados profissionais específicos para formação de EPA, a fim de implantar a prática no país.

### Limitações do estudo

Este estudo apresenta algumas limitações que devem ser consideradas. Primeiramente, embora a pesquisa tenha sido amplamente divulgada em todos os estados brasileiros e tenha alcançado o cálculo amostral necessário, a amostra pode não representar completamente todos os enfermeiros. Em segundo lugar, o estudo baseou-se em respostas auto-relatadas, o que pode levar a um viés de desejabilidade social. Além disso, a ausência de participantes em alguns domínios específicos da PAE, como Pesquisa e Publicação, sugere a possibilidade de uma compreensão incompleta do escopo total, de forma que enfermeiros que poderiam se alinhar com os critérios de definição de PAE podem não ter sido alcançados para participar.

### Contribuições para a Área da Enfermagem

Ao investigar as práticas de enfermeiros em diferentes estados brasileiros, nosso estudo contribui com uma visão mais abrangente das competências e atividades realizadas por esses profissionais, especialmente nas áreas de Cuidados Abrangentes Diretos e Suporte de Sistemas. Ele evidencia as práticas atuais e as lacunas na PAE no Brasil e fornece um impulso para o reconhecimento formal e a integração mais completa desses profissionais nos diferentes níveis do sistema de saúde. Os resultados fornecem uma base sólida para pesquisas futuras, que podem explorar mais profundamente as áreas de menor atuação identificadas, além de investigar as razões para as lacunas existentes na PAE no Brasil.

## CONCLUSÕES

A partir dos nossos resultados, podemos afirmar que os itens avaliados são homogêneos e que a escala mede consistentemente aquilo que se propõe a mensurar, estando, portanto, apta a ser utilizada para mensurar a ocorrência de PAE no país. Ter uma escala validada com essa finalidade é importante para o desenvolvimento do estado da arte da Enfermagem brasileira e oferece evidências científicas para análise da prática profissional, visando à produção de conteúdo na temática da PAE, ainda incipiente no país.

Deste modo, recomendam-se investigações para identificar esses profissionais, ampliar as pesquisas para outros cenários de atuação além da APS e estimular os órgãos legisladores e instituições formadoras de recursos humanos de enfermagem, a fim de superar o desafio de implementação da PAE.
